# Comments on “Emergency Medicine Resident Rotations Abroad: Current Status and Next Steps”

**DOI:** 10.5811/westjem.2016.5.30700

**Published:** 2017-01-20

**Authors:** Gabrielle A. Jacquet, Scott G. Weiner, Janis P. Tupesis

**Affiliations:** * Boston University, Department of Emergency Medicine, Boston, Massachusetts; † Brigham and Women’s Hospital, Department of Emergency Medicine, Boston, Massachusetts; ‡ University of Wisconsin, Madison, Department of Emergency Medicine, Madison, Wisconsin

To the Editor:

Morris and Schroeder have highlighted the need for a uniform and comprehensive national education program for emergency medicine residents doing international rotations. As faculty for a newly released course, *The Practitioner’s Guide to Global Health*, we wanted to call your attention to this innovative resource for preparing resident physicians, medical students, and other trainees to participate in safe and sustainable global health rotations.

In response to the need Morris and Schroeder emphasized, global health faculty from many countries and specialties came together to create a series of open-access, online, timeline-based, interactive modules that 1) prepare medical students, resident physicians, and fellows to safely and effectively participate in global health rotations and projects, 2) permit flexible, asynchronous learning, and 3) provide an electronic evaluation tool for program leadership.

*The Practitioner’s Guide to Global Health* is a three-part timeline-based, interactive, evaluative, open-access course that prepares students and trainees to safely and effectively participate in global health learning experiences. The course is free-of-charge and generates a certificate (upon successful completion) that can be shared with program directors to help facilitate a standardized preparation for trainees across the world. The course is available at tinyurl.com/globalhealthedx.

The three parts of the course are as follows:

Part 1: The Big Picture (to be completed 6–12 months in advance) covers several important “big picture” questions: Why do you want to have a global health learning experience? What kind of experience is right for you and your current level of training? When would be a good time? Where should you do it? How will you fund it?
Part 2: Preparation & On The Ground (to be completed 1–3 months in advance) covers the logistics of planning, security, transportation, communication, personal, health, academic; health: vaccinations and prophylaxis; cultural awareness and sensitivity; packing; logistics and cultural awareness on the ground; and dealing with unexpected situations on the ground.
Part 3: Reflection (to be completed toward the end of your rotation or on your way home) helps you prepare to return, contains important information about dealing with unexpected feelings and health issues, and helps you plan for future work and sustainability.

Several academic institutions and residency programs now require this course for their trainees participating in global health rotations. We hope that this course will be adopted as the national standard for emergency medicine global health training.

